# Ninety-Day Cost, Mortality and Hospital Disparities in Ischemic Stroke: Real-World Evidence from a Czech Administrative Database

**DOI:** 10.3390/healthcare14081056

**Published:** 2026-04-16

**Authors:** Marian Rybář, Gleb Donin, Vojtěch Kamenský, Martina Holá

**Affiliations:** Department of Biomedical Technology, Faculty of Biomedical Engineering, Czech Technical University in Prague, 160 00 Prague, Czech Republic; rybarma5@student.cvut.cz (M.R.);

**Keywords:** ischemic stroke, healthcare costs, 90-day mortality, thrombectomy, provider variability, administrative data

## Abstract

**Background:** Stroke remains a significant health and economic challenge both globally and in the Czech Republic. Although a structured network of specialized stroke centres exists, comparative data on patient outcomes and healthcare costs across hospital types are still lacking in the Czech context. This study analyzed real-world administrative data to assess 90-day mortality and healthcare costs after ischemic stroke, categorized by intervention and provider type. **Methods:** Claims data from six Czech health insurance companies, covering approximately 44% of the population, were used for the years 2017–2020. Patients aged 18 and older with a primary diagnosis of ischemic stroke (ICD-10 code I63) were included. Interventions were categorized as thrombectomy, thrombolysis, or other treatment, and providers were classified as comprehensive stroke centres (CSCs), primary stroke centres (PSCs), secondary referral hospitals (SRHs), or others. Costs were calculated from the payer perspective using administrative claims data, and standardized 90-day mortality and effective cost per survivor (ECPS) were computed. Funnel plots were used to evaluate provider variability in outcomes and costs. The analysis included 23,568 patients (47% female; mean age 70.6). Results: Thrombectomy was associated with the highest mean costs (€13,385), the highest 90-day mortality (29.3%), and the highest ECPS (€18,880). Patients receiving other treatments had the lowest costs (€2725) and lower mortality (14.4%). CSCs recorded the highest average costs (€5087) and mortality (16.7%), while SRHs had the lowest costs (€2204) and mortality (13.7%). Funnel plots revealed greater variability in costs, mainly driven by primary hospitalization, while mortality rates showed less variation. **Conclusions:** These findings suggest that while stroke outcomes are relatively consistent across providers, costs differ, possibly reflecting efficiency differences and case-mix severity. The study is limited by the lack of clinical severity data, highlighting the need to link administrative data with clinical registries for more comprehensive future evaluations.

## 1. Introduction

Stroke is one of the leading causes of death and disability worldwide, representing a major health and economic burden. According to the Global Burden of Disease study by the Institute for Health Metrics and Evaluation, stroke ranked as the second leading cause of death and the third leading cause of combined death and disability globally in 2019 [1], confirming trends already reported in 2017 [2,3]. In Europe, the economic burden of cerebrovascular diseases is substantial, accounting for an estimated €76 billion annually in the European Union in 2021, according to a study published in the European Heart Journal [4]. Given this high burden, there is a clear need to better understand the cost-effectiveness of early interventions in stroke care and to develop standardized methods for benchmarking treatment strategies and healthcare provider performance across regions and hospital systems.

In the Czech Republic, stroke remains a critical public health issue. In 2019, there were 22,203 new cases of stroke and 11,530 stroke-related deaths reported [1]. When broken down by stroke type, ischemic strokes accounted for 16,974 cases and 8951 deaths, while haemorrhagic strokes (intracerebral and subarachnoid combined) accounted for 5229 cases and 2579 deaths [1]. The incidence rate in the Czech Republic was 208.2 new stroke cases per 100,000 inhabitants, with ischemic strokes comprising the majority at 159.1 cases per 100,000 [1,5]. This is higher than the overall European incidence rate of 189.6 per 100,000 but lower than the Central European rate of 261.0 per 100,000. Interestingly, mortality rates in the Czech Republic are slightly better than the European average and significantly better than those reported for Central Europe [1,5].

Acute stroke interventions such as thrombolysis and thrombectomy are recognized as key determinants of survival and cost outcomes. Treatment decisions depend not only on patient factors such as stroke type and severity but also on hospital resources, equipment, and clinical expertise. Guidelines from the American Heart Association and American Stroke Association emphasize the role of specialized stroke centres in improving patient outcomes [6]. In the Czech Republic, evaluations of stroke-centre capabilities have been conducted [7], but prior research has not integrated treatment costs or long-term outcomes such as post-stroke mortality.

Given the complexity of care and the substantial variation in hospital infrastructure, a more granular evaluation of patient outcomes and healthcare costs is warranted. Specifically, analyses that account for intervention type and provider classification at the level of individual patients are essential for benchmarking and assessing quality. The present study aims to investigate differences in 90-day mortality and healthcare costs following ischemic stroke across hospital types in the Czech Republic. By linking these outcomes to intervention strategies and care settings, we seek to better understand efficiency and performance in stroke care delivery.

While previous studies have described stroke epidemiology and outcomes in the Czech Republic, there is limited evidence integrating real-world cost data with mortality outcomes at the patient level using nationwide administrative data. Such analyses are essential for understanding resource utilization, identifying variability across providers, and supporting health system planning and benchmarking. Importantly, administrative data allow for large-scale evaluation of healthcare delivery, although they are limited in capturing clinical detail such as stroke severity.

## 2. Materials and Methods

Administrative claim data from six Czech health insurance companies, covering approximately 44% of all insured persons, were retrieved from a central database maintained by the Health Insurance Bureau in Prague and comprised individual patient claim records from 1 January 2017 to 2 October 2020. The data were processed and operationalised to create patient records comprising information on stroke type, treatment, and survival. The period in which costs and mortality were evaluated was 90 days from day one of the primary hospitalization for acute stroke. The acute stroke was operationalised as the first record of a main ICD10 diagnosis I63. Patients younger than 18 years of age were excluded from the analysis.

Three types of treatment were analyzed in the study: (i) mechanical thrombectomy (hereinafter thrombectomy); (ii) alteplase for the treatment (hereinafter thrombolysis); (iii) treatment other than thrombectomy or thrombolysis (hereinafter other treatment).

In this study, “other treatment” refers to patients who did not receive reperfusion therapy (thrombolysis or thrombectomy) and were instead treated with standard medical care, including supportive care and secondary prevention.

Healthcare providers were divided into 4 types: (i) comprehensive stroke centres (CSCs); (ii) primary stroke centres (PSCs); (iii) Stroke-Ready Hospitals (SRHs; hospitals that are not a CSC or PSC); and (iv) other providers. More detailed information about network of specialized centres for the treatment of stroke in the Czech Republic is described by Mikulík et al. [6].

Costs were derived from administrative claims data submitted by healthcare providers and reimbursed by health insurance funds. These costs reflect payer expenditures, including hospitalization, outpatient care, pharmaceuticals, and medical materials. The analysis therefore represents a costing approach from the payer perspective based on routinely collected data rather than a bottom-up micro-costing methodology. Hospitalization costs were further divided into primary hospitalization costs for acute stroke and secondary hospitalization costs, which captured the costs of hospitalizations other than the primary hospitalization. Secondary hospitalization was defined as any hospitalization, typically a subsequent one, regardless of diagnosis, occurring between day 1 of the primary acute hospitalization and 90 days thereafter. Only patients whose primary hospitalization occurred within the study period and no later than 90 days before its end were included in the analysis. If a patient had more than one primary hospitalization during the study period, the earliest one was selected for analysis, and day 1 of that hospitalization was considered the index date of the study cohort.

As the aim was to compare the impact of the primary acute care provided, patients were assigned to the provider who reported the primary hospitalization, regardless of whether subsequent care was provided by the same or a different provider. Providers with fewer than 30 stroke patients reported as primary care within the study period were excluded from the analysis to check for consistency and reliability of the data. Along with the costs of hospitalization, the costs of outpatient care reported within a period of 90 days from the date of admission for the primary hospitalization were analyzed. Ninety-day crude mortality was calculated as the death of a patient from any cause within 90 days from the date of acute primary admission to hospital. The standardization was performed by adjusting for age as proposed by the Agency for Healthcare Research and Quality U.S. Department of Health and Human Services. The reference population consisted of all examined cases of all examined healthcare providers in the Czech Republic. Individual standardized mortality was the probability of death expressed as 0 to 1.

The primary evaluated outcomes were the total 90-day costs and the 90-day mortality of patients following the day one of the primary acute care admission for ischemic stroke. Furthermore, costs are also presented separately for the primary hospitalization, costs of other hospitalization and outpatient costs. The overall results are presented separately for the evaluated interventions and for individual types of healthcare providers. For individual interventions and types of healthcare providers, the ECPS (effective cost per survivor) indicator was calculated as follows in Equation (1):

(1) $$ECPS=\frac{\sum_{i=1}^{N}C_{i}}{N-D},$$

where *C_i_* is the total 90-day cost of the *i*-th patient, *N* is the total number of patients, and *D* is the number of patients who died within 90 days from the date of admission of the primary hospitalization.

In order to better graphically identify extreme values among healthcare providers, the so-called funnel plots were created as proposed by Spiegelhalter [8]. For standardized 90-day mortality, standardized ratios of monitored events were chosen based on the same author [9]. The plots show the 95% and 99.8% limits of statistical significance.

## 3. Results

In total, the data of 23,568 patients with a mean age of 70.6 years (SD 12.4) who met the inclusion criteria were analyzed (Table 1). There were 11,076 female patients (47%). The highest number of patients, 18,219, were treated with a different treatment than thrombectomy or thrombolysis, 4042 underwent thrombolysis, and 1307 underwent thrombectomy. The mean length of stay (LOS) during primary hospitalization was 8.8 days and appeared relatively homogeneous across treatment groups with a slightly longer stay of 9.1 days in those thrombectomized (Table 1).

In total 10,885 patients were treated in the PSC (46%) followed by the CSC with 8159 (35%) and the SRH with 4066 (17%) patients. Only 458 (1.9%) patients were treated by other healthcare providers.

Table 2 shows the costs of providing healthcare according to the analyzed interventions together with 90-day mortality and the ECPS indicator. Mean patient age and mean duration of primary hospitalization are comparable between interventions. The highest total 90-day costs (EUR 13,385) with the highest 90-day mortality (29.3%) are in the thrombectomy group, where the ECPS value is also the highest (EUR 18,880). On the contrary, we can see the lowest values in the group treated with a different treatment than thrombectomy or thrombolysis, where 90-day costs are EUR 2725, mortality 14.4% and ECPS EUR 3188. These differences should be interpreted with caution, as treatment groups are not directly comparable and reflect differences in stroke severity, clinical indication, and patient characteristics.

**Figure 1:**
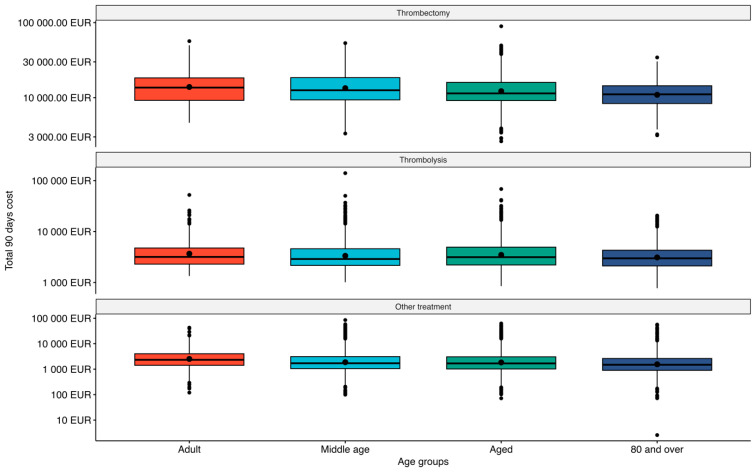
Total 90-day costs for ischemic stroke patients by type of intervention.

Figure 1 shows the distribution of the total 90-day costs for the evaluated interventions, where we can see the variability of these costs more closely. For better visualization, costs are displayed on the logarithmic *y*-axis.

The breakdown of costs into cost of primary hospitalization, cost of other hospitalization and outpatient cost is in Table 3. In the table, the large variability of the costs (based on the presented standard deviation) as well as the difference between the average and median value can be seen.

Table 4, similarly to Table 2, shows the costs together with the 90-day mortality and the ECPS indicator divided by type of healthcare provider. The average age of patients and the average duration of primary hospitalization are also comparable between providers. The highest total 90-day costs (EUR 5087) with the highest 90-day mortality (16.7%) are found for CSCs, where the ECPS value is also the highest (EUR 6093). The lowest cost and ECPS can be seen in the others group, where the costs are EUR 1954 (ECPS EUR 2348), but the mortality rate is the second highest (15.4%). The lowest mortality together with the second lowest costs are for SRHs (13.7% and EUR 2204) with an ECPS value of EUR 2558.

**Figure 2:**
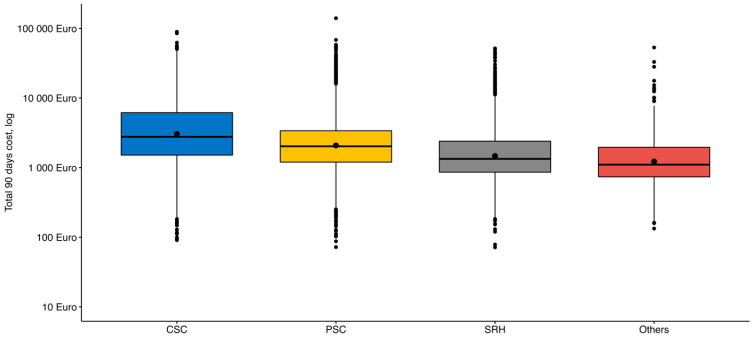
Total 90-day costs for ischemic stroke patients by type of provider.

Figure 2 shows the distribution of the total 90-day costs by type of healthcare provider (costs are again presented on the logarithmic *y*-axis).

**Figure 3:**
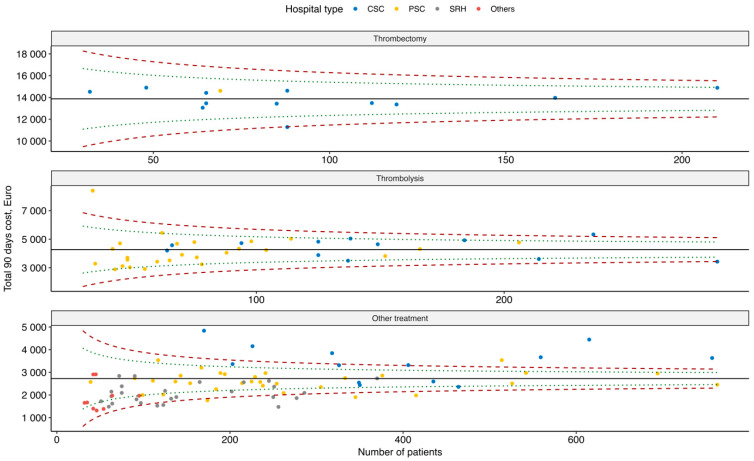
Funnel plot—total 90-day costs by type of provider and type of intervention.

For the evaluated interventions, the funnel plot in Figure 3 shows the 90-day costs in relation to the number of treated patients. Individual types of providers are in the figure distinguished by colours. The figure also shows the 95% and 99.8% control limits (green and red dotted lines, respectively), which identify providers whose costs are statistically significantly different from the national reference value. For thrombectomy, the differences are the smallest, where only one provider was at the upper limit of the 95% interval (higher costs) and one at the lower limit of the 99.8% interval (lower costs). For thrombolysis, and especially for other treatment than thrombectomy or thrombolysis, the differences between providers are more significant. For treatment other than thrombectomy or thrombolysis, we can see that CSC providers have higher costs (above the upper limits of the intervals) and SRHs and other providers are rather below the national reference value or at the lower limits of the intervals.

In the Supplementary Material are funnel plots for the costs of primary (Figure S1) and other hospitalization (Figure S2), where it can be seen that the greatest variability between providers is due to the cost of primary hospitalization.

**Figure 4:**
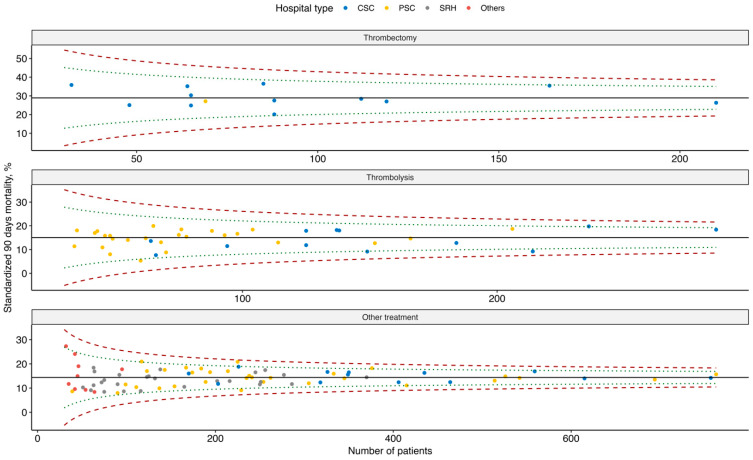
Funnel plot—standardized 90-day mortality by type of provider and type of intervention.

Figure 4 contains funnel plots of the evaluated interventions showing the relationship between standardized 90-day mortality and the number of treated patients. Compared to the funnel plots for costs, there are no healthcare providers that have values above the 99.8% interval and only a minimum of providers is outside the 95% interval. We can thus observe smaller differences between healthcare providers in standardized mortality.

## 4. Discussion

No study was found in the Czech Republic that simultaneously assessed costs and mortality in patients after ischaemic stroke based on administrative data.

The herein-presented study used administrative data from health insurance companies to analyze the costs and mortality of patients hospitalized with ischemic stroke. Analysis of administrative data in the assessment of acute ischemic stroke mortality was also used by Anand et al. [9], who examined data from a private health insurance company between 2012 and 2018 (in total they had data on 117,834 patients) in the USA. Administrative data on acute ischemic stroke and thrombolytic therapy were examined in 2015 in Italy [10]. Another two Italian studies compared general risk-standardized mortality among Lombardy regional hospitals [11] and health outcomes in acute myocardial infarction between national regions [12].

In the Czech Republic, two studies evaluated the costs of rehabilitation treatment on a defined cohort of patients after stroke in a CSC [13,14].

Comprehensive big-data epidemiology evaluations in stroke patients were based on data from the Czech registry of hospitalized patients [15,16,17]. The authors validated the data in this registry and demonstrated the utility of these types of reported data for assessing the incidence of stroke. The evaluation was performed based on data analysis of patients with a reported diagnosis of stroke according to ICD10 (I60, I61, I63, I64, or G45). Given the limitations of the hospital registry, authors did not further evaluate the mortality in these patients and did not examine the type of intervention provided nor the outcomes of the care provided.

The study complements the previous findings by evaluating the costs and mortality of hospitalized patients undergoing different acute interventions as obtained from the administrative data collected from health insurance companies in the Czech Republic.

In our sample of patients, the largest proportion (18,219; 77%) did not undergo thrombectomy or thrombolysis. Although mortality was very similar to that observed in patients who underwent thrombolysis, intervention costs were substantially lower across all monitored categories. The highest intervention costs (mean EUR 13,853; median EUR 11,586) were observed in the thrombectomy group, which also showed the highest standardized 90-day mortality (29.3%). However, these findings should be interpreted with caution because of the heterogeneous patient cohort. As stated in the limitations of the study, administrative data do not capture stroke severity or patients’ functional status. Importantly, the observed differences in mortality and costs between treatment groups should not be interpreted as causal effects of the interventions themselves. Patients undergoing thrombectomy or thrombolysis typically present with more severe strokes and different clinical profiles, which are not captured in administrative data. Therefore, treatment type should be interpreted as a proxy for clinical indication rather than an independent explanatory factor. Furthermore, the dataset does not include information on in-hospital complications, which are known to significantly influence both mortality and costs. The primary contribution of this study is therefore descriptive, providing insight into the distribution of costs and outcomes within the stroke care system rather than evaluating the effectiveness of specific interventions. As shown in the results, the highest number of thrombectomies was performed in CSCs, where patients with more severe forms of ischemic stroke are likely concentrated. The difference in costs was most apparent in primary hospitalization costs; however, the costs of other hospitalizations and outpatient care were also higher than in other evaluated interventions, mainly due to higher material consumption.

The density distribution of total costs within 90 days after the event shows that costs were more variable in patients receiving treatment other than thrombectomy or thrombolysis than in the other intervention groups. A similar pattern was observed for primary hospitalization costs, where the variability was even greater.

The system of care for patients with stroke in the Czech Republic was described recently [8]. When comparing costs according to the type of healthcare provider, the highest total 90-day costs (average EUR 5087) can be seen in patients treated in CSCs while the variability of costs is the highest here, both from the perspective of total costs or from the perspective of the costs of primary hospitalization only. In the case of PSCs, the composition of the total costs is different, the highest part of the costs is the cost of other hospitalizations. The same trend can also be observed in the case of SRH and other healthcare providers.

The difference between the costs of treatment in individual types of healthcare providers is due to differences in the indicated type of treatment. A proportion of 88% of thrombectomy cases were performed in CSCs, 11% in PSCs, and practically not performed in other types of facilities. Likewise, thrombolysis was performed in a CSC or PSC in 97% of cases. Similar conclusions can be found in another local study, showing a great heterogeneity in the volume of thrombolytic care provided between providers in the Czech Republic [7]. It has also been shown elsewhere that the vast majority of recanalization care is provided in certified facilities (CSC or PSC) [8]. Obviously, expertise in the treatment of stroke plays an important role in treatment decisions [8]. Another important factor is the catchment area of the hospital; hospitals with a small catchment area perform fewer thrombolytic interventions.

Comparing individual regions of the Czech Republic, similar variability can be seen with standardized 90-day mortality ranging from 13.9% to 18.6%. The total 90-day costs were varied from EUR 2188 to EUR 4638. These differences can be due to the different distribution of the evaluated healthcare providers in the Czech Republic (especially CSCs and PSCs). The highest costs are in regions with the highest number of CSCs and PSCs. It seems that the fewer specialized centres are in a region the higher the mortality. The differences in mortality between regions are also confirmed by the data published by the Institute of Health Information and Statistics of the Czech Republic [18].

Given the commonly observed skewness of healthcare cost data, there is frequent debate in the literature as to whether the mean is an appropriate estimate of a centre of a dataset [19]. The costs of interventions are therefore presented as median costs, and Figure 1 and Figure 2 show the costs on a logarithmic axis. The median expressing the middle value of an ordered set of values is frequently a more adequate alternative to mean [20]. However, with regard to the objectives of a cost analyses such as improving financial effectiveness between methods, centres, etc., the total costs are important. These can be evaluated and compared to any defined benchmark using averages rather than individual values or medians. Therefore, even with regard to the distorted distribution of the cost data, it is recommended to use mean values for the sake of comparative cost analyses [19,21,22]. This can be well supported by another stroke studies presenting their results as average costs [12,23,24,25,26,27,28].

The skewness of the data also affects the presented result in the form of ECPS. The ECPS indicator was first proposed for the analysis of the cost-effectiveness of intensive care in the 1990s [29,30]. Subsequently, its use was extended to other areas of healthcare. In recent years, this indicator has reappeared in the professional literature [12,31,32,33,34]. The ECPS combines information on the costs incurred and the “effect” of healthcare, i.e., patient survival. However, the ECPS is also a ratio indicator, and its statistical interpretation is therefore quite non-trivial. For this reason, it can be concluded that ECPS can play the role of a supporting indicator of the quality of healthcare provision, but not a basic one.

Funnel plots provide a detailed view of the differences in the results of individual healthcare providers. Funnel plots were designed as a replacement for so-called caterpillar plots, i.e., graphs showing indicator values and related confidence intervals. Since 2005, the use of funnel plots has expanded considerably even in the professional literature [35,36,37,38,39,40,41,42,43,44,45,46]. Currently, funnel plots are used for the presentation of quality indicators in healthcare, e.g., in Great Britain. British authorities state that this type of plot provides better information for evaluating the quality of healthcare providers. The construction of the funnel plot is based on several assumptions and estimates regarding the behaviour of statistical variables. As part of this analysis, basic scenarios were used, facilitating the identification of extreme values of costs and mortality among providers.

An additional limitation is that the administrative data do not provide detailed clinical information on stroke severity, and we cannot assess patients’ functional status when evaluating healthcare outcomes [47]. Other studies encountered similar limitations of administrative data [10,11]. It would be highly desirable to link administrative data with clinical patient-level data. This can be done in the following analysis, where it is possible to link part of the data with the data in the Registry for Stroke Care Quality (RES.Q) [46] and evaluate care outcomes other than mortality (e.g., functional status, quality of life). These data could further be used to simulate the longer-term impacts and costs of ischemic stroke.

Data from six of the seven health insurance companies in the Czech Republic were analyzed, covering approximately 44% of insured persons. Although the authors assume that this did not introduce major bias, it should still be considered a limitation of the study.

Despite these limitations, administrative data are a valuable source of information for assessing costs associated with disease treatment and evaluating healthcare outcomes such as mortality.

## 5. Conclusions

This study provides a large-scale overview of healthcare costs and 90-day mortality among patients with ischemic stroke using administrative data from six health insurance companies in the Czech Republic. Differences observed between treatment groups and provider types likely reflect variations in patient characteristics, stroke severity, and care pathways rather than direct effects of specific interventions.

Administrative data represent a valuable resource for understanding resource utilization and system-level variation in stroke care. These data can support health system planning, benchmarking across providers, and identification of areas for improvement. Future studies should aim to link administrative datasets with clinical registries to enable more robust risk adjustment and outcome evaluation.

## Figures and Tables

**Table 1:** Basic characteristics of the patient group.

Characteristics	Total N = 23,568	Thrombectomy N = 1307	Thrombolysis N = 4042	Other Treatment N = 18,219
Sex, (%)				
Female	11,076 (47%)	660 (50%)	1854 (46%)	8562 (47%)
Male	12,492 (53%)	647 (50%)	2188 (54%)	9657 (53%)
Age, mean (SD)	70.6 (12.4)	68.9 (12.9)	69.7 (13.1)	70.9 (12.2)
Type of provider *, (%)				
CSC	8159 (35%)	1147 (88%)	1834 (45%)	5178 (28%)
PSC	10,885 (46%)	146 (11%)	2107 (52%)	8632 (47%)
SRH	4066 (17%)	13 (1.0%)	99 (2.4%)	3954 (22%)
Others	458 (1.9%)	1 (<0.1%)	2 (<0.1%)	455 (2.5%)
LOS in days, mean, (SD)	8.8 (7.3)	9.1 (7.9)	8.8 (7.9)	8.8 (7.1)

Note: *—according to primary hospitalization, CSC = comprehensive stroke centre, PSC = primary stroke centre, SRH = Stroke-Ready Hospital, SD = standard deviation.

**Table 2:** Mortality and costs of individual interventions in patients with ischemic stroke.

Intervention	Number of Patients	Mean Age	LOS, Mean	St. 90-Day Mortality	Total 90-Day Costs, Mean, EUR	Cost of Primary Hosp., Mean, EUR	ECPS, EUR
Thrombectomy	1307	68.9	9.1	29.3%	13,385	9243	18,880
Thrombolysis	4042	69.7	8.8	14.9%	4248	2476	4970
Other treatment	18,219	70.9	8.8	14.4%	2725	1285	3188

Note: ECPS = Effective cost per survivor, LOS = length of stay, St. = standardized.

**Table 3:** The cost of interventions in patients with ischemic stroke according to type of cost.

	Total N = 23,568	Thrombectomy N = 1307	Thrombolysis N = 4042	Other Treatment N = 18,219
Total 90-day cost, EUR				
Mean (SD)	3603 (4915)	13,853 (7705)	4248 (4515)	2725 (3752)
Median [IQR]	2062 [1170; 3808]	11,586 [9088; 16,310]	3038 [2179; 4666]	1661 [1006; 2974]
Costs of primary hosp., EUR				
Mean (SD)	1931 (3067)	9243 (5154)	2476 (2428)	1285 (2154)
Median [IQR]	1029 [546; 1911]	8482 [6774; 10,868]	1920 [1490; 2602]	791 [467; 1349]
Costs of other hosp., EUR				
Mean (SD)	837 (2722)	2335 (4602)	948 (3173)	704 (2378)
Median [IQR]	0 [0; 743]	633 [0; 2524]	0 [0; 993]	0 [0; 557]
Outpatient costs, EUR				
Mean (SD)	836 (1611)	2275 (3390)	824 (1457)	735 (1323)
Median [IQR]	443 [180; 858]	465 [153; 1505]	481 [205; 905]	433 [176; 830]

Note: hosp. = Hospitalization, SD = standard deviation, IQR = interquartile range.

**Table 4:** Mortality and costs of ischemic stroke by type of healthcare provider.

Type of Provider	Number of Patients	Mean Age	LOS, Mean	St. 90-Day Mortality	Total 90-Day Costs, Mean, EUR	Cost of Primary Hosp., Mean, EUR	ECPS, EUR
CSC	8159	70.2	8.77	16.7%	5087	2967	6093
PSC	10,885	70.6	9.00	14.7%	3083	1565	3613
SRH	4066	71.0	8.57	13.7%	2204	940	2558
Others	458	72.8	8.24	15.4%	1954	953	2348

Note: CSC = Comprehensive stroke centre, PSC = primary stroke centre, SRH = Stroke-Ready Hospital, ECPS = effective cost per survivor, St. = standardized.

## Data Availability

Data is unavailable due to its large extent and the provider’s policy on the provision of administrative data held by a third party.

## Supplementary Materials

The following supporting information can be downloaded at https://www.mdpi.com/article/10.3390/healthcare14081056/s1, Figure S1: Funnel plot—primary hospitalization costs by type of provider and type of intervention; Figure S2: Funnel plot—other hospitalization costs by type of provider and type of intervention.

## Abbreviations

The following abbreviations are used in this manuscript:

AHRQ	Agency for Healthcare Research and Quality
CSC	Comprehensive Stroke Centre
ECPS	Effective Cost per Survivor
GDPR	General Data Protection Regulation
ICD-10	International Classification of Diseases, 10th Revision
IQR	Interquartile Range
LOS	Length of Stay
PSC	Primary Stroke Centre
SRH	Stroke-Ready Hospital

## References

[1] Feigin V.L., Stark B.A., Johnson C.O., Roth G.A., Bisignano C., Abady G.G., Abbasifard M., Abbasi-Kangevari M., Abd-Allah F., Abedi V. (2021). Global, Regional, and National Burden of Stroke and Its Risk Factors, 1990–2019: A Systematic Analysis for the Global Burden of Disease Study 2019. Lancet Neurol..

[2] Kyu H.H., Abate D., Abate K.H., Abay S.M., Abbafati C., Abbasi N., Abbastabar H., Abd-Allah F., Abdela J., Abdelalim A. (2018). Global, Regional, and National Disability-Adjusted Life-Years (DALYs) for 359 Diseases and Injuries and Healthy Life Expectancy (HALE) for 195 Countries and Territories, 1990–2017: A Systematic Analysis for the Global Burden of Disease Study 2017. Lancet.

[3] Krishnamurthi R.V., Ikeda T., Feigin V.L. (2020). Global, Regional and Country-Specific Burden of Ischaemic Stroke, Intracerebral Haemorrhage and Subarachnoid Haemorrhage: A Systematic Analysis of the Global Burden of Disease Study 2017. Neuroepidemiology.

[4] Luengo-Fernandez R., Walli-Attaei M., Gray A., Torbica A., Maggioni A.P., Huculeci R., Bairami F., Aboyans V., Timmis A.D., Vardas P. (2023). Economic Burden of Cardiovascular Diseases in the European Union: A Population-Based Cost Study. Eur. Heart J..

[5] Powers W.J., Rabinstein A.A., Ackerson T., Adeoye O.M., Bambakidis N.C., Becker K., Biller J., Brown M., Demaerschalk B.M., Hoh B. (2019). Guidelines for the Early Management of Patients with Acute Ischemic Stroke: 2019 Update to the 2018 Guidelines for the Early Management of Acute Ischemic Stroke: A Guideline for Healthcare Professionals from the American Heart Association/American Stroke Association. Stroke.

[6] Mikulík R., Václavík D., Šaňák D., Bar M., Ševčík P., Kalita Z., Wahlgren N. (2010). A Nationwide Study on Topography and Efficacy of the Stroke Treatment Network in the Czech Republic. J. Neurol..

[7] Mikulik R., Bar M., Cernik D., Herzig R., Jura R., Jurak L., Neumann J., Sanak D., Ostry S., Sevcik P. (2021). Stroke 20 20: Implementation Goals for Intravenous Thrombolysis. Eur. Stroke J..

[8] Spiegelhalter D.J. (2005). Funnel Plots for Comparing Institutional Performance. Stat. Med..

[9] Anand S.K., Benjamin W.J., Adapa A.R., Park J.V., Wilkinson D.A., Daou B.J., Burke J.F., Pandey A.S. (2021). Trends in Acute Ischemic Stroke Treatments and Mortality in the United States from 2012 to 2018. Neurosurg. Focus.

[10] Berta P., Seghieri C., Vittadini G. (2013). Comparing Health Outcomes among Hospitals: The Experience of the Lombardy Region. Health Care Manag. Sci..

[11] Francisci S., Gigli A., Gesano G., Folino-Gallo P. (2008). Decomposing Differences in Acute Myocardial Infarction Fatality in Italian Regions. Health Care Manag. Sci..

[12] Angerová Y., Maršálek P., Chmelová I., Gueye T., Barták M., Uherek Š., Bříza J., Rogalewicz V. (2021). Cost Analysis of Early Rehabilitation after Stroke in Comprehensive Cerebrovascular Centres in the Czech Republic. Cent. Eur. J. Public Health.

[13] Kratochvílová A., Rogalewicz V., Angerová Y., Gueye T., Maršálek P., Chmelová I., Barták M. (2021). Early Rehabilitation after Stroke in Comprehensive Cerebrovascular Centres in the Czech Republic: A Comparison of Three Stroke Units. Kontakt.

[14] Sedova P., Brown R.D., Zvolsky M., Belaskova S., Volna M., Baluchova J., Bednarik J., Mikulik R. (2021). Incidence of Stroke and Ischemic Stroke Subtypes: A Community-Based Study in Brno, Czech Republic. Cerebrovasc. Dis..

[15] Sedova P., Brown R.D., Zvolsky M., Kadlecova P., Bryndziar T., Kubelka T., Weiss V., Volný O., Bednarik J., Mikulik R. (2017). Incidence of Hospitalized Stroke in the Czech Republic: The National Registry of Hospitalized Patients. J. Stroke Cerebrovasc. Dis..

[16] Sedova P., Brown R.D., Zvolsky M., Kadlecova P., Bryndziar T., Volny O., Weiss V., Bednarik J., Mikulik R. (2015). Validation of Stroke Diagnosis in the National Registry of Hospitalized Patients in the Czech Republic. J. Stroke Cerebrovasc. Dis..

[17] Demozem2020.Pdf. https://www.uzis.cz/res/f/008370/demozem2020.pdf.

[18] Thompson S.G., Barber J.A. (2000). How Should Cost Data in Pragmatic Randomised Trials Be Analysed?. BMJ.

[19] Bang H., Zhao H. (2014). Cost-Effectiveness Analysis: A Proposal of New Reporting Standards in Statistical Analysis. J. Biopharm. Stat..

[20] Diehr P., Yanez D., Ash A., Hornbrook M., Lin D.Y. (1999). Methods for Analyzing Health Care Utilization and Costs. Annu. Rev. Public Health.

[21] Zhou X.H., Melfi C.A., Hui S.L. (1997). Methods for Comparison of Cost Data. Ann. Intern. Med..

[22] Mayer S.A., Copeland D., Bernardini G.L., Boden-Albala B., Lennihan L., Kossoff S., Sacco R.L. (2000). Cost and Outcome of Mechanical Ventilation for Life-Threatening Stroke. Stroke.

[23] Smeds M., Skrifvars M.B., Reinikainen M., Bendel S., Hoppu S., Laitio R., Ala-Kokko T., Curtze S., Sibolt G., Martinez-Majander N. (2022). One-Year Healthcare Costs of Patients with Spontaneous Intracerebral Hemorrhage Treated in the Intensive Care Unit. Eur. Stroke J..

[24] Ding R., Zhu D., Ma Y., Shi X., He P. (2020). Comparison of Health Service Use and Costs in Stroke with and without Comorbidities: A Cross-Sectional Analysis Using China Urban Medical Claims Data. BMJ Open.

[25] Dodel R.C., Haacke C., Zamzow K., Pawelzik S., Spottke A., Rethfeldt M., Siebert U., Oertel W.H., Schöffski O., Back T. (2004). Resource Utilization and Costs of Stroke Unit Care in Germany. Value Health.

[26] Xu X.M., Vestesson E., Paley L., Desikan A., Wonderling D., Hoffman A., Wolfe C.D.A., Rudd A.G., Bray B.D. (2018). The Economic Burden of Stroke Care in England, Wales and Northern Ireland: Using a National Stroke Register to Estimate and Report Patient-Level Health Economic Outcomes in Stroke. Eur. Stroke J..

[27] Luengo-Fernandez R., Violato M., Candio P., Leal J. (2020). Economic Burden of Stroke across Europe: A Population-Based Cost Analysis. Eur. Stroke J..

[28] Smithies M.N., Bihari D., Chang R. (1994). Scoring Systems and the Measurement of Icu Cost Effectiveness. Reanim. Urgences.

[29] Chalfin D.B., Cohen I.L., Lambrinos J. (1995). The Economics and Cost-Effectiveness of Critical Care Medicine. Intensive Care Med..

[30] Kulkarni A., Divatia J. (2013). A Prospective Audit of Costs of Intensive Care in Cancer Patients in India. Indian J. Crit. Care Med..

[31] Kortelainen S., Curtze S., Martinez-Majander N., Raj R., Skrifvars M.B. (2022). Acute Ischemic Stroke in a University Hospital Intensive Care Unit: 1-Year Costs and Outcome. Acta Anaesthesiol. Scand..

[32] Efendijev I., Folger D., Raj R., Reinikainen M., Pekkarinen P.T., Litonius E., Skrifvars M.B. (2018). Outcomes and Healthcare-Associated Costs One Year after Intensive Care-Treated Cardiac Arrest. Resuscitation.

[33] Raj R., Bendel S., Reinikainen M., Hoppu S., Laitio R., Ala-Kokko T., Curtze S., Skrifvars M.B. (2018). Costs, Outcome and Cost-Effectiveness of Neurocritical Care: A Multi-Center Observational Study. Crit. Care.

[34] Abramo G., D’Angelo C.A., Grilli L. (2015). Funnel Plots for Visualizing Uncertainty in the Research Performance of Institutions. J. Informetr..

[35] Noyez L. (2009). Control Charts, Cusum Techniques and Funnel Plots. A Review of Methods for Monitoring Performance in Healthcare. Interact. Cardiovasc. Thorac. Surg..

[36] Kunadian B., Dunning J., Roberts A.P., Morley R., de Belder M.A. (2009). Funnel Plots for Comparing Performance of PCI Performing Hospitals and Cardiologists: Demonstration of Utility Using the New York Hospital Mortality Data. Catheter. Cardiovasc. Interv..

[37] Willik E.M., Zwet E.W., Hoekstra T., Ittersum F.J., Hemmelder M.H., Zoccali C., Jager K.J., Dekker F.W., Meuleman Y. (2021). Funnel Plots of Patient-reported Outcomes to Evaluate Health-care Quality: Basic Principles, Pitfalls and Considerations. Nephrology.

[38] Seaton S.E., Manktelow B.N. (2012). The Probability of Being Identified as an Outlier with Commonly Used Funnel Plot Control Limits for the Standardised Mortality Ratio. BMC Med Res. Methodol..

[39] Ieva F., Paganoni A.M. (2015). Detecting and Visualizing Outliers in Provider Profiling via Funnel Plots and Mixed Effect Models. Health Care Manag. Sci..

[40] Verburg I.W., Holman R., Peek N., Abu-Hanna A., de Keizer N.F. (2018). Guidelines on Constructing Funnel Plots for Quality Indicators: A Case Study on Mortality in Intensive Care Unit Patients. Stat. Methods Med. Res..

[41] Dover D.C., Schopflocher D.P. (2011). Using Funnel Plots in Public Health Surveillance. Popul. Health Metr..

[42] Solomon P.J., Kasza J., Moran J.L., The Australian and New Zealand Intensive Care Society (ANZICS) Centre for Outcome and Resource Evaluation (CORE) (2014). Identifying Unusual Performance in Australian and New Zealand Intensive Care Units from 2000 to 2010. BMC Med. Res. Methodol..

[43] Spiegelhalter D.J. (2005). Handling Over-Dispersion of Performance Indicators. Qual. Saf. Health Care.

[44] Rousson V., Le Pogam M.-A., Eggli Y. (2018). Control Limits to Identify Outlying Hospitals Based on Risk-Stratification. Stat. Methods Med. Res..

[45] Spiegelhalter D., Sherlaw-Johnson C., Bardsley M., Blunt I., Wood C., Grigg O. (2012). Statistical Methods for Healthcare Regulation: Rating, Screening and Surveillance: Statistical Methods for Healthcare Regulation. J. R. Stat. Soc. Ser. A (Stat. Soc.).

[46] Registr RES-Q. https://www.cmp.cz/registr-res-q.

[47] Baldereschi M., Balzi D., Di Fabrizio V., De Vito L., Ricci R., D’Onofrio P., Di Carlo A., Mechi M.T., Bellomo F., Inzitari D. (2018). Administrative Data Underestimate Acute Ischemic Stroke Events and Thrombolysis Treatments: Data from a Multicenter Validation Survey in Italy. PLoS ONE.

