# Supplementary material for: Ninety-Day Cost, Mortality and Hospital Disparities in Ischemic Stroke: Real-World Evidence from a Czech Administrative Database
Source: Healthcare (Basel). 2026 Apr 16;14(8):1056. doi: 10.3390/healthcare14081056 (PMC13116848; doi:10.3390/healthcare14081056)
Supplement: Supplementary file 1 [file healthcare-14-01056-s001.zip › healthcare-4177661-supplementary.pdf]

Supplementary material

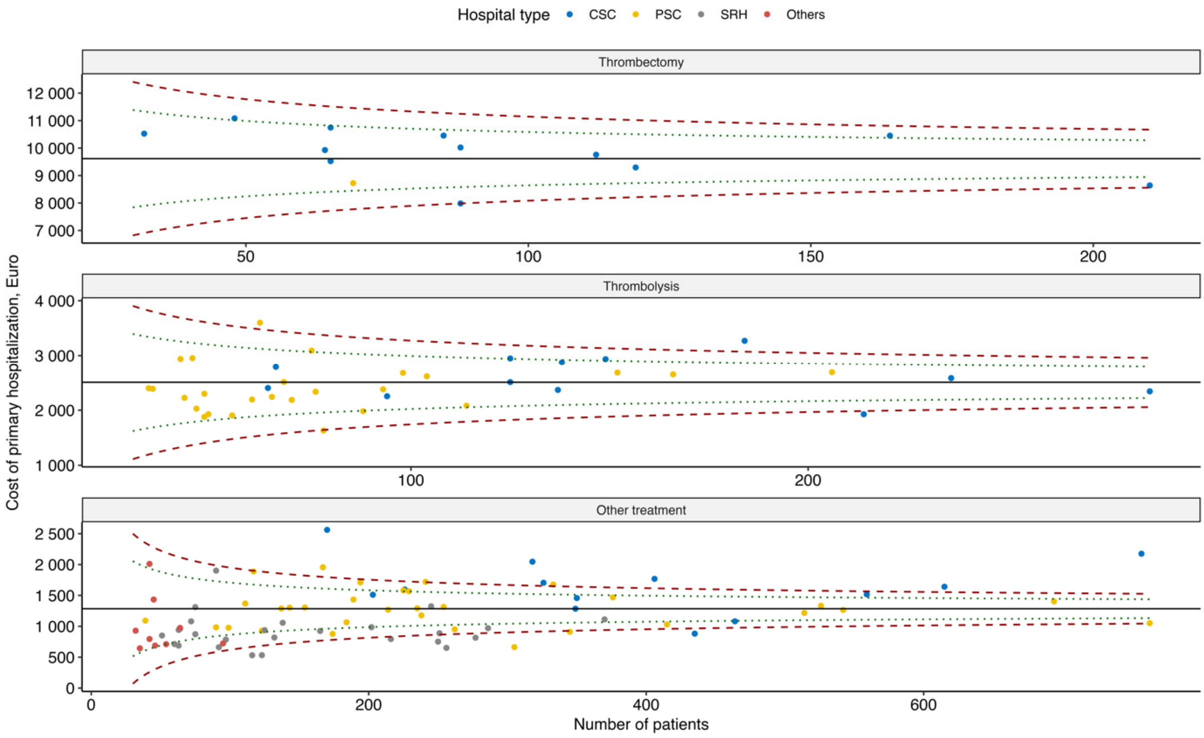

Figure S1: Funnel plot – Primary hospitalization costs by type of provider and type of intervention

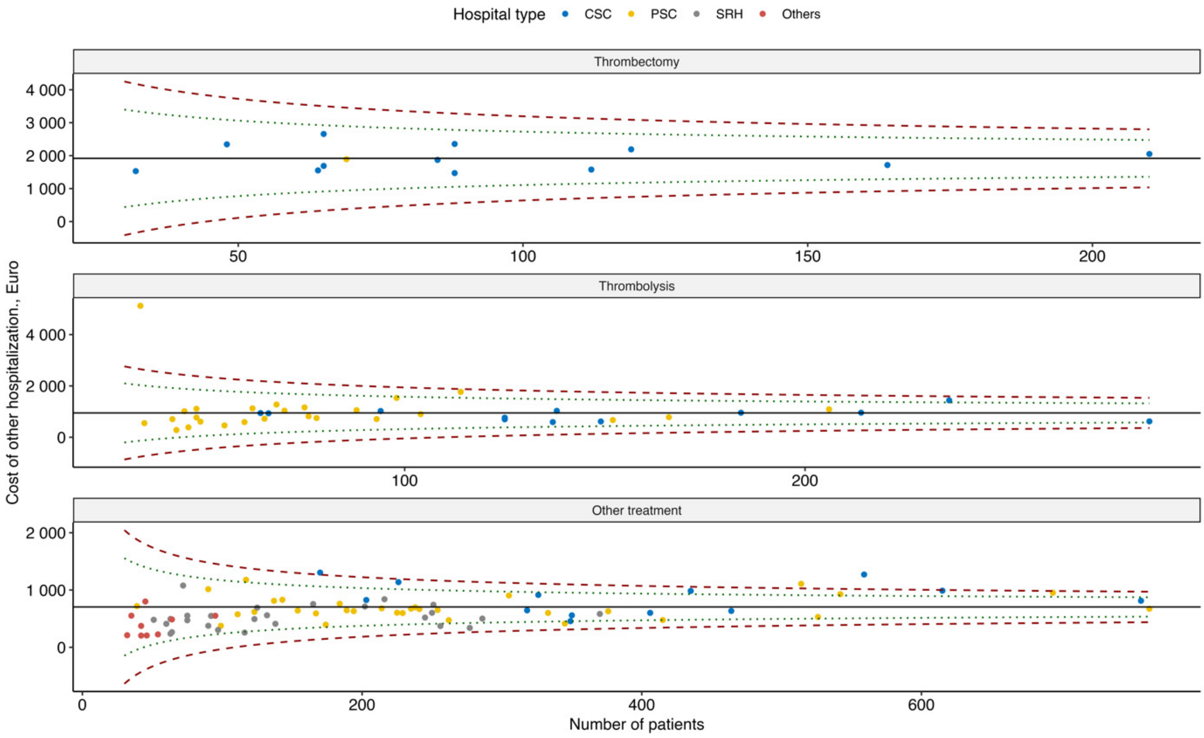

Figure S2: Funnel plot – Other hospitalization costs by type of provider and type of intervention
